# Recurrent Meningitis After a Post-Traumatic Intradiploic Arachnoid Cyst: A Case Report

**DOI:** 10.7759/cureus.37124

**Published:** 2023-04-04

**Authors:** Kivanc Yangi, Mehmet Yasar Kaynar

**Affiliations:** 1 Neurological Surgery, Prof. Dr. Cemil Tascioglu City Hospital, Istanbul, TUR; 2 Neurological Surgery, Istanbul University-Cerrahpasa, Cerrahpasa Medical Faculty, Istanbul, TUR

**Keywords:** csf-culture negative meningitis, recurrent meningitis, post-traumatic intradiploic arachnoid cyst, intradiploic arachnoid cyst, negative csf culture

## Abstract

Intradiploic arachnoid cysts are infrequent but benign lesions of the central nervous system. Etiologically, they can be non-traumatic or post-traumatic in origin. We present an unusual case of a post-traumatic intradiploic arachnoid cyst presented with recurrent meningitis episodes. A 68-year-old female patient was admitted to the emergency department with fever and loss of consciousness, with a history of cranial operation due to a gunshot injury to the left occipital bone 45 years ago. On the patient's initial examination, nuchal rigidity was detected; Kernig's and Brudzinski's signs were positive. A lumbar puncture has been performed, and the patient is diagnosed with meningitis. The patient had been admitted to the emergency department with rhinorrhea after a minor blunt head trauma six years ago. As we understood from the patient's medical records, a couple of millimetric non-specified pneumocephalus areas, located next to the sella turcica, were detected on the cranial non-contrast-enhanced CT scan after the minor blunt trauma to the frontal bone. However, there was no sign of any obvious skull base fracture. The patient was hospitalized for five days and discharged on the sixth day without any complaints. After the discharge, the patient was admitted to other hospitals five times in the last five years with fever and anxiety. On all her admissions, the patient was diagnosed with CSF-culture-negative meningitis and treated with different unknown antibiotics. Magnetic resonance imaging (MRI) showed some irregularities and thinning at the inner table of the left occipital bone; there was an enlargement of the diploic distance of the occipital bone on the left side. MR cisternography showed cerebrospinal fluid (CSF) fistulizing areas just below the thickened and irregular part of the occipital bone. CSF fistula was communicated with the left lateral ventricle. The occipital horn of the left lateral ventricle was enlarged. We performed a surgical repair in order to cover the defective areas of the occipital and mastoid bones. The retromastoid approach was used. Pedunculated muscle flaps to cover the defective bony areas are used and secured with fibrin glue. There is no evidence of recurrence during the one-year follow-up period of the patient. We present this unusual case to emphasize that if post-traumatic intradiploic arachnoid cysts remain untreated, severe complications, such as episodes of recurrent meningitis, may occur. Although a few cases of these cysts are reported in the literature, a case of post-traumatic intradiploic arachnoid cyst presenting with recurrent meningitis has not been reported. In patients with recurrent meningitis, when no prominent etiology is found and if there is a trauma to the related bone in the patient's history, post-traumatic intradiploic arachnoid cyst should be included in the differential diagnosis.

## Introduction

Arachnoid cysts are benign pathologies that generally exist in the intracranial region. These cysts seldomly develop in intradiploic space. Dunkser and McCreary defined these cysts as “leptomeningeal cysts” [1]. The term “intradiploic arachnoid cyst” was first described by Weinand et al. [2], when cranial arachnoid cysts developed as folded extensions of the arachnoid membrane through minor defects in the dura mater, eroded through the inner table, expanded within the diploe, and eroded the outer table of the skull [2]. Cerebrospinal fluid accumulation within the diploic space is extremely rare [3]. Intradiploic cerebrospinal fluid (CSF) collections can occur due to trauma but can also be non-traumatic. Rarely, these cysts may also appear when there is no trauma in the patient's history. On the other hand, generally, intradiploic arachnoid cysts, or leptomeningeal cysts, occur after a head trauma or a cranial surgical intervention [3]. In the literature, these cysts are called “intradiploic CSF fistula”, “intraosseous leptomeningeal cysts”, and traumatic or post-traumatic “intradiploic arachnoid cysts”. We prefer to use the term “post-traumatic arachnoid cyst” since it defines the etiology better. However, the terminology is confused in the literature since the terms “intraosseous leptomeningeal cysts” and “intradiploic CSF fistula” are used for both non-traumatic and post-traumatic intradiploic arachnoid cysts [3]. Post-traumatic intradiploic arachnoid cysts are rare but usually benign conditions. Although it is a benign disease that does not need to be treated [4], early surgical exploration should be considered when it becomes symptomatic. In the case of delayed treatment, undesirable severe complications may develop. We present a 68-year-old female patient with a post-traumatic intradiploic arachnoid cyst in the left occipital bone. The patient presented with recurrent meningitis episodes.

## Case presentation

A 68-year-old female patient was admitted to the emergency department with fever and loss of consciousness. According to her son's statement, she was drowsy at home. The son called the ambulance to take her to the hospital. When the past medical records were examined, it was revealed that the patient had been operated on for penetrating head trauma after a gunshot injury to the left occipital bone, 45 years ago. No medical records could have been reached about the details of the previous surgery. However, it could have been understood from the partially fused craniotomy defects on the CT scan that the patient had been operated on with left occipital craniotomy. The radiology specialists of our hospital also verified this information. Long after this injury, she was admitted to the hospital after minor blunt head trauma six years ago, with rhinorrhea. As we understood from the patient's medical records, a couple of millimetric non-specified pneumocephalus areas, located next to the sella turcica, were detected on the cranial non-contrast-enhanced CT scan after the minor blunt trauma to the frontal bone. However, there was no sign of any obvious skull base fracture. The patient was hospitalized for five days, treated with the required antibiotics, and discharged on the sixth day without any complaints.

**Figure 1:**
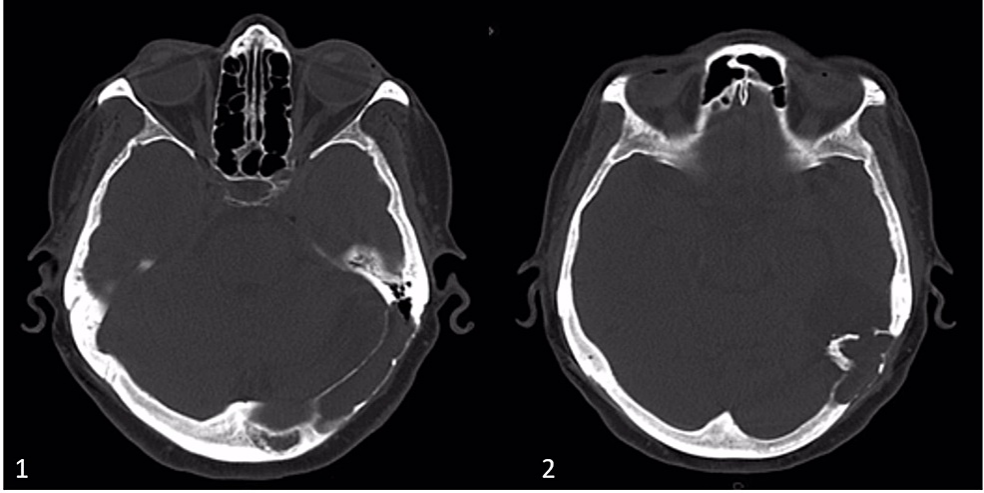
Preoperative axial CT scans showing lesions in the diploe of petrous and occipital bones.

**Figure 2:**
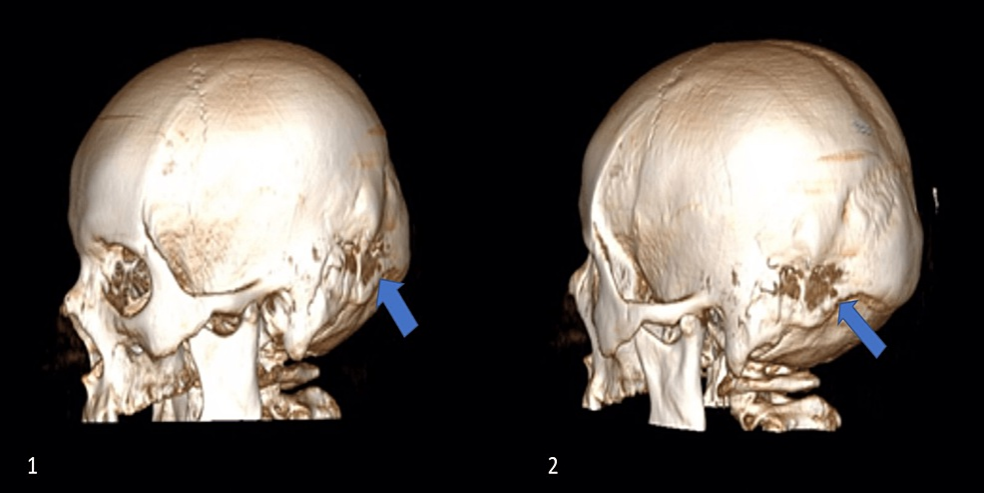
Preoperative three-dimensional reconstructed CT scan showing the left occipital bone defect (Blue arrows).

**Figure 3:**
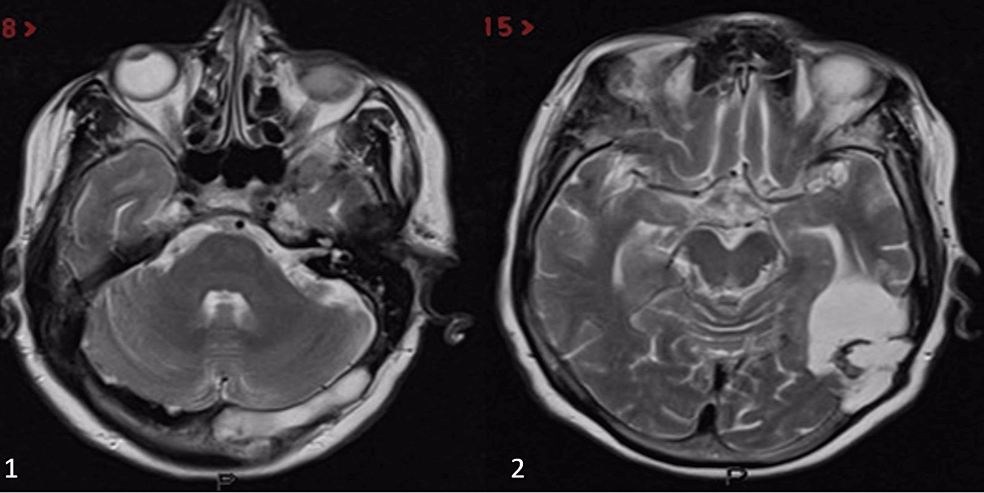
Preoperative axial T2-weighted MRI showing enlargement of the diploic distance extending towards the temporal bone. There was a CSF leakage at the opening of the mastoid cells. CSF: Cerebrospinal fluid

Beginning from the patient's discharge from the hospital six years ago, five CSF-culture-negative meningitis episodes were found in the patient's history. Different unknown antibiotic therapies treated her past meningitis attacks. Her current detailed physical examination showed hepatomegaly and nuchal rigidity; Kernig's and Brudzinski's signs were positive and GCS (Glasgow Coma Scale) score was 15 out of 15. Meningitis was diagnosed with a lumbar puncture on the patient's last admission to the hospital. Her CSF analysis results were: a glucose level of 35 mg/dl, a protein level of 469 mg/dl, and a white blood cell count was found 3239 per microliter, corresponding blood glucose level was 80 mg/dl. Empirical antibiotic therapy was started with vancomycin 1 gr, two doses per day, and meropenem 500 mg, three doses per day, for 14 days. Computerized tomography (CT) of the cranium showed a lesion in the diploe of the petrous bone (Figure 1), with cortical irregularities and thickening in the anterior part of the left occipital bone, which was extending towards the intracranial region. A three-dimensional reconstructed CT scan showed a left occipital bone defect (Figure 2, with blue arrows). The CT scan revealed an area of encephalomalacia in front of the bony spur, which is related to lateral ventricles. Contrast-enhanced magnetic resonance imaging (MRI) showed irregularities in the inner table of the left occipital bone and enlargement in the diploic distance of the left half of the occipital bone, and thinning of the inner table (Figure 3). Enlargement of the diploic distance extends towards the temporal bone and causes some CSF deposits at the opening of the mastoid cells and encephalomalacia in the left cerebellar hemisphere and left temporal and occipital lobes. Magnetic resonance (MR) cisternography showed CSF leakage below the area of cortical thickness and irregularities related to the lateral ventricle. The occipital horn of the left lateral ventricle was enlarged. We performed surgical repair to cover the bony defects and repair the areas of the cerebrospinal fluid leakage. After the linear retromastoid skin incision, suboccipital muscle is dissected laterally, and irregular bony areas are explored. A widened left occipital craniotomy is performed. As soon as the bone is detected, cerebrospinal fluid accumulation is drained. The fistula tract is disconnected from the left lateral ventricle. The defects at the diploic distance of the inner table of the left occipital bone and the mastoid bone are covered by pedunculated muscle flaps, which are taken from suboccipital muscles. The pedunculated muscle flaps are covered with fibrin glue. A negative CSF culture had been reported. The patient had consulted the microbiology and infectious diseases department; however, the responsible organism could not be clarified after the analysis. The infectious diseases department suggested continuing with the empirical antibiotic treatment. Empirical antibiotic therapy had completed in 14 days. The patient was discharged on the fourteenth postoperative day without any complaints. There was neither evidence of recurrent meningitis nor CSF fistulas in the patient's one-year follow-up period.

## Discussion

Intradiploic arachnoid cyst was first proposed by Dunkser and McCreary in 1971 [1]. Diploic space may be an additional space for CSF reabsorption, as in some cases in the literature [5]. Intradiploic CSF collections have been given different names, such as leptomeningeal cysts and post-traumatic intradiploic arachnoid cysts, rare variants of growing skull fractures [3].

These cysts can originate in the related bone's inner table and may cause a tear on the dura mater. This situation is ended up with an accumulation of CSF covered by an arachnoid membrane within the diploic space [6].

Non-traumatic intradiploic arachnoid cysts should also be considered in the differential diagnosis. They are generally found incidentally. No treatment is necessary unless they become symptomatic [4]. Extension of the arachnoid diverticulum into the diploic space, presumably because of a minor congenital disability of the dura mater, may be the cause of non-traumatic cases [7]. The other reason is the trauma to the skull. Although it can be detected in any part of the skull, post-traumatic intradiploic arachnoid cyst is generally seen in the parietal bone. That is probably because temporal and occipital bones have overlying musculature to protect them [8]. The exploratory surgery was decided upon the patient's choice and related to the symptoms in different reports in the literature [7,9]. However, early exploratory surgery is recommended in cases with symptoms. Surgical repair with muscle flaps or pericranial grafts, water-tight dural closure, and securement with fibrin glue is usually the treatment of the traumatic cysts, according to the literature [3].

CT or MRI can help assess the size, content, and extension of the intradiploic CSF collections. The hypodense intradiploic lesion can be detected on a CT scan like a normal CSF appearance. MR imaging study should be performed to distinguish the lesion from the dermoid and epidermoid cyst [7]. Although the outer table of the bone is generally expanded but not interrupted, the inner table may be interrupted. It may provoke the passing of CSF; therefore, a connection between the posterior fossa subarachnoid space and the intradiploic cyst occurs.

We do not know the main reason behind meningitis in our case since there was a closed fistula. The patient had no open CSF fistula. The reason behind meningitis, in this case, is presumably CSF leakage into the mastoid air cells.

Surgery is the primary treatment option for post-traumatic intradiploic arachnoid cysts in symptomatic cases. The purpose of the surgery is the repair of the dural lacerations and the correction of the bone. Therefore pedunculated muscle flaps, fibrin glue, and galea grafts can be used for water-tight dural closure, and cranioplasty [10] can secure the bone flap. If these cysts remain untreated, they may cause severe complications such as recurrent meningitis, as seen in our case.

## Conclusions

Post-traumatic intradiploic arachnoid cysts are benign and rare lesions of the central nervous system. Although benign in nature, if left untreated, they may cause complications such as headaches, intracranial hypotension, and even meningitis. Early surgical treatment should be performed to prevent severe complications.
